# Are monocyte-to-HDL and C-reactive protein-to-albumin ratios useful for the diagnosis and follow-up of Takayasu arteritis?

**DOI:** 10.1590/1806-9282.20231683

**Published:** 2024-05-17

**Authors:** Elif Altunel Kılınç, Gizem Varkal, Gizem Kırmızıer, İpek Türk, Hüseyin Turgut Elbek Özer

**Affiliations:** 1 Çukurova University, Faculty of Medicine, Department of Rheumatology – Adana, Turkey.

**Keywords:** Takayasu arteritis, Inflammation mediators, Acute-phase proteins

## Abstract

**OBJECTIVE:**

In this study, we aimed to investigate the role of erythrocyte sedimentation rate, C-reactive protein, neutrophil/lymphocyte ratio, platelet/lymphocyte ratio, monocyte/lymphocyte ratio, red blood cell distribution width, mean platelet volume, monocyte/HDL ratio, and C-reactive protein/albumin ratio in the diagnosis and treatment follow-up of active and remission Takayasu arteritis patients compared with healthy control group.

**METHODS:**

This is a retrospective case-control study in which 56 Takayasu arteritis patients and 40 age- and sex-matched healthy control were included. The blood values of Takayasu arteritis patients were analyzed during their active period and post-treatment remission periods, after comparing them with the healthy control. Furthermore, all parameters were evaluated by receiver operating characteristic analysis.

**RESULTS:**

Erythrocyte sedimentation rate, C-reactive protein, neutrophil/lymphocyte ratio, platelet/lymphocyte ratio, monocyte/lymphocyte ratio, monocyte/HDL ratio, and C-reactive protein/albumin ratio values were significantly higher in active Takayasu arteritis patients compared with healthy control and remission Takayasu arteritis groups. In the receiver operating characteristic analysis performed in active Takayasu arteritis and Takayasu arteritis patients in remission, C-reactive protein had the highest power to indicate disease activity, followed by C-reactive protein/albumin ratio, erythrocyte sedimentation rate, and monocyte/HDL ratio. When Takayasu arteritis in remission was compared with the healthy control, a significant difference was found between erythrocyte sedimentation rate, C-reactive protein, red blood cell distribution width, and C-reactive protein/albumin ratio, while no significant difference was found between monocyte/HDL ratio values.

**CONCLUSION:**

C-reactive protein/albumin ratio and red blood cell distribution width can be used in the diagnosis of Takayasu arteritis, and C-reactive protein/albumin ratio, red blood cell distribution width, and monocyte/HDL ratio measurements can be used in the follow-up. As C-reactive protein/albumin ratio is more powerful than C-reactive protein in differentiating the Takayasu arteritis group from the healthy control group, evaluation of C-reactive protein/albumin ratio together with albumin instead of evaluation of C-reactive protein alone when diagnosing the disease may help us to obtain more accurate results in daily practice.

## Medical Subject Headings

1. Arteritis, Takayasu
2. Acute phase proteins
3. Vasculitis

## INTRODUCTION

Takayasu arteritis (TA) is a chronic inflammatory disease of unknown etiology that mainly targets the aorta and its main branches^1^. Acute phase reactants (APRs) and imaging methods are helpful in the diagnosis^2^. Disease activity indices largely utilize C-reactive protein (CRP) and erythrocyte sedimentation rate (ESR) for both clinical and acute phase responses. However, CRP and ESR are not always elevated in acute TA disease. High concentrations of APRs are usually observed during active TA, but some studies have observed normal APRs in 10–30% of patients. This leads to the search for markers to be used both at the time of diagnosis and in treatment follow-up^3,4^.

Acute phase reactants are proteins that show a 25% increase (positive APR) or decrease (negative APR) in serum concentration in response to inflammation^5^. Neutrophil/lymphocyte ratio (NLR), platelet/lymphocyte ratio (PLR), monocyte/lymphocyte ratio (MLR), red blood cell distribution width (RDW), mean platelet volume (MPV), monocyte/HDL ratio (MHR) and CRP/albumin ratio (CAR) are the parameters currently investigated.

In this study, we aimed to investigate the roles of NLR, PLR, MLR, RDW, MPV, RDW, and MPV, especially CAR and MHR in the diagnosis, disease activity, and follow-up of TA, which we think may be an alternative to APRs such as ESR and CRP, which are currently used but are not definitive indicators of disease activity and inflammation.

## METHODS

This is a retrospective case-control study. It included 56 patients with newly diagnosed TA between January 2007 and 2023 according to the classification criteria determined by the American College of Rheumatology (ACR) in 1990. Forty healthy individuals of similar age and sex were also included^6^. Activation of the patients was analyzed by Kerr score, and patients with Kerr score >2 were included. These patients were considered active TA^4^, who were all evaluated with blood results during remission periods after treatment. In patients with previously active disease, remission was defined as more than 6 months of stable disease without the development of new vascular lesions^7^. Patients under the age of 18 years and patients with active infection, malnutrition, malignancy, pregnancy, proteinuria, chronic liver disease, chronic renal failure, autoimmune disease, hematological disease, or lymphoproliferative history were excluded. This study protocol was approved by the Human Research Ethics Committee of Çukurova University (No. 137/2023).

The hospital’s electronic medical record system was utilized to collect demographic information, clinical characteristics, pharmacological history, laboratory results, and imaging findings of the aorta and its branches. The analyses were conducted on ESR, CRP, NLR, PLR, MLR, RDW, MPV, MHR, and CAR values of the TA patients during the active and remission phases. The hematological parameters were evaluated by taking peripheral blood samples from the patients in a tube with EDTA. It was obtained that ESR for the patients aged under 50 years was 0–15 mm/h, ESR for women was 0–20 mm/h, ESR for men aged over 50 years was 0–20 mm/h, ESR for women was 0–30 mm/h, CRP was 0–5 mg/L, RDW was 11.8–13.4%, MPV was 6.5–11.6 fL, and albumin was 3.4–5.4 g/dL and they were considered normal laboratory values. NLR was obtained by dividing the number of neutrophils into lymphocytes, PLR by dividing the platelet into lymphocytes, MLR by dividing the monocyte to lymphocyte, MHR by dividing the number of monocytes by HDL concentration, and CAR by dividing CRP by albumin. In age and gender-matched healthy control (HC) group, ESR, CRP, NLR, PLR, MLR, RDW, MPV, MHR, and CAR values were accessed from the hospital electronic system. Laboratory parameters of active TA, TA in remission, and HC group were evaluated.

Receiver operating characteristic (ROC) analysis of inflammation markers between active TA and TA in remission groups was performed. The most powerful parameters in showing disease activation were CRP, CAR, ESR, and MHR, respectively (p<0.05, for all). For CAR, sensitivity was 80%, specificity was 95%, and area under the curve (AUC) (95%CI) was 0.916 (0.863–0.969), and for MHR, sensitivity was 64%, specificity was 85%, and AUC (95%CI) was 0.808 (0.729–0.887).

### Statistical analysis

The statistical analyses were conducted using the SPSS for Windows 25.0 software. The normality of the variables was assessed through both visual and analytical methods (Kolmogorov-Smirnov/Shapiro-Wilk tests). A p-value greater than 0.05 in the Kolmogorov-Smirnov test indicated that the data followed a normal distribution. When normal distribution was not determined, the patient and control groups were compared using the Mann-Whitney U test. The median values were taken. Wilcoxon signed-rank test was used to compare the values of a single dependent group in two-time intervals when there was no normal distribution. The chi-square test was used for the group comparisons of the qualitative variables. The AUC, sensitivity, specificity, and cutoff values were compared using the ROC curve. The Spearman correlation test was used for the correlation evaluation, and the correlation coefficient was taken as rho. The correlation coefficient<0.25 means no relationship or very weak relationship, 0.25–0.5 means weak to moderate relationship, 0.5–0.75 means strong relationship, and>0.75 means very strong relationship. Statistically, p<0.05 was considered significant.

## RESULTS

A total of 56 patients with the TA (87.5% female, at the mean age of 40.5±10.08 years) and 40 age-sex matched healthy in the control group (82.5% female, at the mean age of 38.03±7.85 years) were included in the study. No statistically significant difference was found in the age and gender distribution of the patient and HC group (p=0.149 and p=0.499, respectively) (Table 1).

**Table 1:** Demographic data of Takayasu arteritis and healthy control group.

		Takayasu arteritis (n=56)	Healty control group (n=40)	p-value
Gender	Female	49	33	0.499
	Male	7	7	
Age (years±SD)		40.5±10.08	38.03±7.859	0.149

Inflammation markers of active TA, healthy population, and TA in remission groups are summarized in Table 2. According to these results, ESR, CRP, NLR, PLR, MLR, RDW, MHR, and CAR values of active TA patients were statistically significantly higher than those of the healthy population (p≤0.001, p≤0.001, p≤0.001, p≤0.001, p=0.016, p≤0.001, p≤0.001, p=0.004, p≤0.001, p≤0.001, and p≤0.001, respectively). There was no statistically significant difference between the MPV results of both groups (p=0.241). In the active TA and remission TA groups, ESR, CRP, NLR, PLR, MLR, MPV, MHR, and CAR values measured in the remission TA group were statistically significantly lower than those of active TA (p<0.001, p<0.001, p<0.001, p<0.001, p<0.001, p=0.030, p=0.001, p=0.027, p<0.001, p<0.001, and p<0.001, respectively). No significant change was found in RDW values (p=0.056) (Table 2). When the TA group in remission was compared with the HC group, ESR, CRP, RDW, and CAR were statistically significant, while no significant difference was found between NLR, PLR, MPV, and MHR (p=0.04, p=0.02, p=0.038, p<0.05, p=0.545, p=0.696, p=0.666, and p=0.687, respectively).

**Table 2:** Evaluation of inflammation markers in active Takayasu arteritis compared with the healthy control group and Takayasu arteritis in remission.

	TA active (n=54) median (IQR)	Healthy control group (n=40) median (IQR)	p-value	Remission (n=54) median (IQR)	p-value
ESR	44 (28.5–62)	7 (4–9)	<0.001	10.5 (5.25–18.75)	<0.001
CRP	14.6 (8.750–40)	2.4 (1.2–3.350)	<0.001	2 (1.125–2.425)	<0.001
NLR	3.375 (2.235–5.4)	2.1 (1.31–3.36)	<0.001	2.2 (1.6025–3.03)	<0.001
PLR	156 (113.5–202.4)	125 (95.5–157)	0.016	127.4 (9.1–162.5)	0.030
MLR	0.37 (0.26–0.52)	0.21 (0.145–0.29)	<0.001	0.25 (0.18–0.35)	0.001
RDW	16.3 (14.4–17.550)	14.75 (13.3–16.1)	0.004	15.8 (13.9–17.3)	0.056
MPV	8.4 (7.6–8.9)	8.7 (8–9.4)	0.241	8.8 (8–9.75)	0.027
MHR	0.0175 (0.01–0.025)	0.0085 (0.005–0.02)	<0.001	0.009 (0.00625–0.011)	<0.001
CAR	3.775 (1.65–6.29)	0.03 (0.02–0.045)	<0.001	0.375 (0.2–0.51)	<0.001

CAR: CRP-to-albumin ratio; CRP: C-reactive protein; ESR: erythrocyte sedimentation rate; MHR: monocyte-to-HDL ratio; MLR: monocyte-to-lymphocyte ratio; MPV: mean platelet volume; NLR: neutrophil-to-lymphocyte ratio; PLR: platelet-to-lymphocyte ratio; RDW: red cell distribution width; TA: Takayasu arteritis.

The ROC analysis was performed to address the question of the predictive ability of inflammation markers by evaluating the active TA group and the HC group. In this analysis, the dependent variable is the presence of TA. The cutoff, sensitivity, and specificity values and AUC results obtained from this analysis are given in Table 3. According to these results, CAR was the most powerful marker among the markers evaluated to indicate inflammation. This was followed by ESR, CRP, MLR, NLR, PLR, RDW, MHR, and PLR, respectively. MPV value was not found to be a reliable marker of inflammation (p=0.275).

**Table 3:** Performance of inflammation markers to discriminate patients with active Takayasu arteritis and healthy controls.

	ESR	CRP	NLR	PLR	MLR	RDW	MHR	CAR
Cutoff	11.5	4.745	2.58	136.5	0.265	15.3	0.01275	0.1275
Sensitivity	91.1	85.7	67.9	57.1	73.2	62.5	64.3	64.3
Specificity	92.5	85	67.5	65	70	6.,5	65	35
AUC (95%CI)	0.948(0.899–0.996)	0.915 (0.859–0.971)	0.730 (0.628–0.832)	0.647 (0.538–0.756)	0.772 (0.673–0.871)	0.692 (0.586–0.799)	0.683 (0.565–0.802)	0.952 (0.9–1)
p-value	<0.001	<0.001	<0.001	0.014	<0.001	0.01	0.002	<0.001

CAR: CRP-to-albumin ratio; CRP: C-reactive protein; ESR: erythrocyte sedimentation rate; MHR: monocyte-to-HDL ratio; MLR: monocyte-to-lymphocyte ratio; MPV: mean platelet volume; NLR: neutrophil-to-lymphocyte ratio; PLR: platelet-to-lymphocyte ratio; RDW: red cell distribution width; TA: Takayasu arteritis.

According to the correlation analysis, ESR was strongly correlated with CRP and CAR (r=0.642 and r=0.576, respectively) and weakly to moderately correlated with RDW and MHR (r=0.459 and r=0.336, respectively). CRP was very strongly correlated with CAR (r=0.828), strongly correlated with ESR and RDW (r=0.624 and r=0.512, respectively), and weakly to moderately correlated with MHR (r=0.307). CAR, which was found to be one of the strongest markers of inflammation by the ROC analysis, was found to be very strongly correlated with CRP and ESR (r=0.828 and 0.576, respectively) and weakly to moderately correlated with RDW (r=0.376). MHR was weakly to moderately correlated with ESR, CRP, and RDW (r=0.336, r=0.307, and r=0.322, respectively) and strongly correlated with MLR (r=0.531).

## DISCUSSION

In this study, we aimed to evaluate the inflammation markers of TA patients in active and remission periods and investigate the place of NLR, PLR, MLR, RDW, and MPV, especially MHR and CAR, which have been the subject of many studies recently, in disease diagnosis and follow-up of TA.

In our study, ESR, CRP, NLR, PLR, MLR, MHR, and CAR were significantly higher in the active TA group than in the HC group and the TA group in remission. In the TA group in remission, ESR, CRP, CAR, and RDW were significantly higher than the HC group. According to ROC analysis, the most powerful parameter in distinguishing TA from the HC group was CAR, followed by ESR, CRP, MLR, NLR, RDW, MHR, and PLR, respectively. CAR was strongly correlated with CRP, which was of course included in the formula, was strongly correlated with ESR, and was weakly to moderately correlated with RDW.

Erythrocyte sedimentation rate and CRP have been the best-known APRs for many years. Except for inflammation, ESR may increase in many inflammation-related conditions such as anemia, pregnancy, and old age^8^. CRP better represents many chronic inflammation-related conditions such as obesity, DM, HT, atherosclerosis, and the risk of cardiovascular heart disease due to atherosclerosis^8,9^. NLR, PLR, MLR, RDW, and MPV are also parameters that have been investigated in many studies on active infection and chronic inflammation^10,11^.

In two previous studies, PLR and NLR levels were found to be higher in the TA patients with high disease activity compared with the healthy population, and these parameters were found to have a positive correlation with disease activation. They also reported that NLR and PLR predicted the TA similarly and that both PLR and NLR were correlated with ESR and CRP. Based on these findings, Pan et al. concluded that PLR and NLR could be used to reflect the inflammatory response and disease activity in the TA, and Li et al. concluded that PLR was more powerful than NLR in detecting the disease activation in their patients^12,13^. In our study, similarly, NLR was found to be significantly higher in the active TA compared with the HC group and in the active TA patients compared with the patients in remission. In PLR, there was no significant difference between the active TA and healthy population, but it decreased significantly when we compared the active period and remission in the same patient. When the formula of these markers was analyzed, it was observed that the difference was platelet-derived. In the study conducted by Pan et al. the blood samples were taken in a tube without anticoagulant and evaluated. In our study, a tube with EDTA was used. We know that EDTA can cause pseudothrombocytopenia^14^. Therefore, we think that the discordance in PLR value can be a negative effect of EDTA tube use. In the study conducted by Li et al. 180 (62.2%) active TA patients were included. In our study, this number was 56. We think that the difference in sample size caused this result^13^.

The recent study on this subject was conducted by Seringec Akkececi et al. in 2019. It was found that ESR, CRP, NLR, PLR, MLR, RDW, and CAR were significantly higher in the active TA patients compared with the control and remission groups. It was reported that CAR had the highest correlation with the disease activity and showed a positive correlation with ESR, CRP, NLR, PLR, MLR, and RDW^7^. In our study, ESR, CRP, NLR, MLR, RDW, and CAR were found to be significantly higher in the patients with active TA compared with healthy subjects, and PLR was not found to be different. Similarly, ESR, CRP, NLR, PLR, MLR, MLR, and CAR decreased in the patients in remission. The results of our study are similar with the study by Seringec Akkececi et al. Unlike this study, we had the opportunity to evaluate MHR.

The limitations of our study include limited patient sample size, single-center design, and retrospective methodology. The majority of the patients included in our study were on steroid therapy after diagnosis. This may have caused changes in hematological parameters in the TA group in remission. To the best of our knowledge, an important aspect of this study is that it is the first research to demonstrate the importance of MHR in diagnosing and indicating disease activation in TA. Furthermore, rather than categorizing patients into two groups based on their activity level, we examined both the periods of active disease and remission within the same patient group. This approach allowed us to assess the parameters that could potentially be valuable in monitoring the treatment progress.

## CONCLUSION

Monocyte/HDL ratio and CAR are usable markers both in the diagnosis of TA and to indicate active disease. Especially as CAR is a stronger marker than CRP in showing inflammation, evaluation of CRP together with albumin in daily practice reaches more accurate results in differentiating inflammation. These markers will give us an advantage, particularly in patients with normal ESR and CRP, but clinical suspicion of activation. NLR, PLR, MLR, and RDW are inexpensive tests available in almost every laboratory and are promising in clinical practice, especially in TA patients with normal CRP and ESR values. In our opinion, especially CAR and MHR can be used as indicators of active inflammation in TA patients. Based on the result of our study that CAR is a more powerful marker than CRP in showing active inflammation, we think that the evaluation of albumin results together with CRP may give more accurate results in evaluating inflammation in daily practice. As clinical studies supporting these data increase, we think that its use in daily practice will become widespread.
